# Embracing the unknown: investigating medical communication around uncertainty and the implications on patient and family well-being

**DOI:** 10.1186/s13023-024-03050-y

**Published:** 2024-02-26

**Authors:** Leisha Devisetti

**Affiliations:** University of California, Berkeley, University Avenue and, Oxford St, 94720 Berkeley, CA USA

## Abstract

Rare diseases present immense challenges to physicians, patients, and the healthcare system at large due to a scarcity of research and knowledge in the field. This contributes to uncertainty surrounding rare diseases, which can hinder the management of these chronic conditions. An analysis of my family’s experience battling my mother’s ameloblastic carcinoma highlights the difficulties in communicating the uncertainty around rare diseases and their damaging effects on our family’s well-being. Here, we will recognize the importance of acknowledging uncertainty during diagnoses and advocating for enhanced detection strategies. The goal of this article is to emphasize that effective medical communication around rare diseases, accessibility to accurate information, proper services, and a shift toward a culture that prioritizes patient well-being are critical for improving health outcomes for rare disease patients.

## Rare diseases

In the United States, a rare disease is defined as a condition affecting fewer than 200,000 individuals [1]. Despite around 7,000 identified rare diseases impacting over 30 million Americans, merely 500 of these conditions have approved treatments and many have limited research due to difficulty finding enough suitable patients for clinical trials [2]. As a result, accessing complete and accurate information, specialized services, and appropriate management for rare diseases is a challenging endeavor.

This scarcity of research and knowledge results in the pervasive uncertainty surrounding symptoms and treatments, exacerbating the troubles associated with managing a chronic condition. Within my own family, I observed how we faced the complexities and uncertainties associated with having a rare disease when my mother was diagnosed with ameloblastic carcinoma—an exceedingly rare odontogenic cancer with only a few hundred reported cases since 1948 [3].

## Uncertainty in rare diseases

Healthcare is inherently a field of uncertainty. Especially in an era of personalized healthcare, it is becoming increasingly complex to apply population-based findings to individuals. Doing so demands an extremely nuanced understanding of the condition, which is often not possible for rare diseases since rare disease cases do not occur as frequently. By virtue of informed consent, physicians are responsible for thoroughly and effectively communicating information. While providers may hesitate due to concerns about overwhelming patients with intricate explanations, studies suggest that expressions of uncertainty actually enhance patient satisfaction [4]. As such, the presumed advantages in suppressing uncertainty pose significant challenges to accurate diagnoses and inhibit gaining valuable insight into rare diseases, presenting a destructive cycle for both patients and physicians.

## The diagnostic odyssey

Diagnosing rare diseases is tricky because symptoms can resemble common conditions or present as seemingly unrelated problems [5]. In 2014, my mother complained of a sensitive left jaw impacting her ability to eat. As a working mother, doctors initially attributed the jaw pain to work-related stress, resulting in a diagnosis of bruxism, or excessive teeth grinding. Over the following three years, she developed migraines, which the doctors quickly linked to work stress once again due to her past medical reports. Unexplained foul-smelling breath and an X-ray in January 2022 revealed deteriorating tooth roots, leading to the extraction of two teeth. The tooth deterioration, swelling, and pain on the left side of her face were dismissed as symptoms of periodontal disease and temporomandibular joint dysfunction, despite the mystery of visibly healthy gums and localized tooth loss.

During this time, my sister and I experienced numerous milestones while my mother struggled with the pain and lack of help with her symptoms. Reflecting on moments such as assisting my sister in moving into her first apartment, my mother described her inability to communicate her jaw pain due to the diagnosis’s limitations. She could only hope that the symptoms would subside by trusting the treatment for periodontal disease. Yet, the emotional burden of feeling unable to care for her family compounded, as she exerted herself balancing work, family, and health. The lack of clear words to express her pain and the perceived simplicity of doctors’ diagnoses led her to minimize the true burden of her symptoms, perpetually disrupting how she handled her health and familial responsibilities.

My mother still persistently advocated for more tests and scans to explain the gaps in the diagnosis. Thus, nearly a year after losing her teeth, a final CT scan and a national effort in analyzing a biopsy sample revealed an ameloblastoma measuring 4.7 × 3.8 × 4.3 cm and extending into the maxillary sinus and orbital floor. The astounding findings led to my mother being diagnosed with Stage II ameloblastoma, a condition that had been destroying her facial bone for over 9 years. Ameloblastoma is generally a slow-growing aggressive tumor that begins in the inner epithelial cells of tooth enamel [6]. However, it has the capacity to turn malignant (into ameloblastic carcinoma) if it goes untreated for a long period of time [6]. We only found out about the malignancy of my mother’s ameloblastoma after receiving the postoperative biopsy results from the surgery in May 2023. Subsequent MRI findings also uncovered remaining portions of the tumor near the skull base, thus requiring a second surgery. Due to the rare nature of the condition, the tumor’s response to radiation therapy was uncertain; nonetheless, my mother underwent radiation therapy until September 2023 as a precautionary measure.

## Importance of communication around uncertainty

The 9-year-long diagnostic odyssey proved detrimental to my mother’s and our family’s physical and mental well-being. While the tumor could have arisen *de novo*, we will never be sure if the lengthy diagnostic process is what contributed to the ameloblastoma turning malignant. For rare disease patients globally, the average diagnosis time is 4 to 5 years, causing considerable harm to their emotional, psychological, and practical well-being, and possibly even worsening their condition beyond return [7]. Patients with rare diseases report higher levels of anxiety due to feeling a lack of control over the disease along with social and functional troubles in explaining symptoms to friends and family, so communication during the diagnosis and treatment process is increasingly important [8–11].

When my mother shared the details of her condition with our family, the true quantity and severity of her symptoms were a shock to us, as she had been suppressing her struggles for such a long time. My mother recounted sleepless nights from both the pain and the fear of not knowing what to do about the physical and emotional burden she felt. She described how she frequently searched what her symptoms meant on Google to attempt to piece together her symptoms on her own. Similar anxiousness is a common theme among rare disease patients, where the absence of a diagnosis leads to emotions of abandonment or contempt and elevated stress levels, creating a heavy reliance on Internet sources to provide more information about the questions left unanswered by physicians [12–14]. The lack of information culminates in rare disease patients who feel isolated, so we must make changes to ensure that initial diagnoses acknowledge the nuances and explicate any inconsistencies in the evidence [9].

Since my mother had not been equipped with the medical terms to explain the extent of her symptoms, my family had trouble understanding the nature of her condition. Learning her true diagnosis helped us support her as a family. We undertook household chores, attended medical appointments together, ensured we were informed about the condition, and actively listened to her concerns to create a supportive environment where she felt cared for. Although challenging for her as a parent, she finally felt empowered to take a break and accept help. The validation for her symptoms provided by a thorough, accurate, and effective diagnosis played a pivotal role in accepting the care she needed from her family. For rare disease patients, having support groups they can trust, both among healthcare professionals and within their families, is exceptionally crucial, so sharing her fears and uncertainty with trusted physicians and our family became vital for preserving my mother’s well-being [15, 16].

## Conclusion

The challenges rare diseases pose for both physicians and patients restrict effective communication about symptoms and treatments. Physicians struggle with limited training and deficits in the available literature on rare diseases, preventing comprehensive care [17]. The healthcare system’s culture also links uncertainty with failure, exacerbating the difficulty of conveying uncertainty to patients [4]. For individuals affected by rare diseases, their diagnoses are frequently communicated using complex medical jargon without the necessary ongoing support available for other chronic conditions, hindering proper communication and profoundly impacting the well-being of these patients and their families [18]. Therefore, promoting effective communication around rare diseases by ensuring the accessibility of adequate information and services within the healthcare system and beyond is of utmost importance.

Cases like that of my mother’s highlight the significance of remaining vigilant for symptoms and advocating for better communication about uncertainties in health. By fostering a medical culture that prioritizes comprehensive evaluation and acknowledgment of symptoms over rushing to a definitive diagnosis, we can improve the overall care for rare disease patients and satisfy individuals’ currently unmet needs.

My family and I have witnessed firsthand how hasty diagnoses, in hopes of bypassing uncertainty, harm patients and families. Thus, in this time of personalized healthcare, we must revolutionize how we communicate uncertainty [4]. We need a comprehensive approach that acknowledges the limitations of our current research, addresses gaps in detection strategies, and invests in patient advocacy groups [19]. Rare disease patients are entitled to accurate information, consistent education, and psychosocial support, so working toward establishing robust relationships between clinicians and patients is essential for supporting the well-being of rare disease patients. Diagnostic communication will empower patients to actively engage in healthcare decision-making and ensure they connect with the most suitable treatments [20]. As a result, we must all be proponents of change that brings effective medical communication around uncertainty to the forefront in the pursuit of better health outcomes.

## Data Availability

Provided in References section of the manuscript (see below).

## Declarations

### Authors’ information (optional)

Leisha Devisetti is an undergraduate student studying at the University of California, Berkeley. When her mother was diagnosed with ameloblastic carcinoma in June 2023, she experienced first-hand the adversities rare disease patients must face. Her family’s experience has augmented her passion for pursuing a career as a physician. She is interested in discovering ways of alleviating patient suffering. As such, while rare disease patients are often impacted in ways that we tend to overlook, Leisha hopes to share her family’s story to raise awareness about the reality behind rare conditions and advocate for positive change.
